# Delineation of agricultural fields in smallholder farms from satellite images using fully convolutional networks and combinatorial grouping

**DOI:** 10.1016/j.rse.2019.111253

**Published:** 2019-09-15

**Authors:** C. Persello, V.A. Tolpekin, J.R. Bergado, R.A. de By

**Affiliations:** Faculty of Geo-Information Science and Earth Observation (ITC), University of Twente, the Netherlands

**Keywords:** Field boundary detection, Semantic edge detection, Image segmentation, Convolutional neural networks, Deep learning, Smallholder farming

## Abstract

Accurate spatial information of agricultural fields in smallholder farms is important for providing actionable information to farmers, managers, and policymakers. Very High Resolution (VHR) satellite images can capture such information. However, the automated delineation of fields in smallholder farms is a challenging task because of their small size, irregular shape and the use of mixed-cropping systems, which make their boundaries vaguely defined. Physical edges between smallholder fields are often indistinct in satellite imagery and contours need to be identified by considering the transition of the complex textural pattern between fields. In these circumstances, standard edge-detection algorithms fail to extract accurate boundaries. This article introduces a strategy to detect field boundaries using a fully convolutional network in combination with a globalisation and grouping algorithm. The convolutional network using an encoder-decoder structure is capable of learning complex spatial-contextual features from the image and accurately detects sparse field contours. A hierarchical segmentation is derived from the contours using the oriented watershed transform and by iteratively merging adjacent regions based on the average strength of their common boundary. Finally, field segments are obtained by adopting a combinatorial grouping algorithm exploiting the information of the segmentation hierarchy. An extensive experimental analysis is performed in two study areas in Nigeria and Mali using WorldView-2/3 images and comparing several state-of-the-art contour detection algorithms. The algorithms are compared based on the precision-recall accuracy assessment strategy which is tolerating small localisation errors in the detected contours. The proposed strategy shows promising results by automatically delineating field boundaries with F-scores higher than 0.7 and 0.6 on our two test areas, respectively, outperforming alternative techniques.

## Highlights

• Deep learning technique to automatically delineate agricultural fields in smallholder farms
• Extensive experimental analysis on two real case studies in Nigeria and Mali
• Precision-recall framework for accuracy assessment introduced in remote sensing
• The proposed technique compares favourably against state-of-the-art algorithms.

## 1 Introduction

Improving the capability to map and monitor the spatial distribution of agricultural resources is crucial for increasing the agricultural production and ensuring food security in many parts of the world (Debats et al., 2016). In Sub-Saharan Africa (SSA), agriculture is dominated by smallholder farms, characterized by rain-fed production for predominantly household consumption. Smallholder farmers cultivate >80% of the cropland available in Africa, employing about 60% of the labour market (Lowder et al., 2016). Among the regions where smallholder farming is predominant, SSA is considered one of the most important because of its geographical size and the potential for growth in the coming decades. However, the large growth in the African population urgently demands increased production and improvements in the governance of food production systems. These improvements are also a prerequisite for realizing the United Nations (UN) Sustainable Development Goals (SDG), and in particular target 2.3, which aims to double the agricultural productivity and the incomes of small-scale food producers by 2030 (UN General Assembly, 2015).

Spatial information of agricultural fields across Africa is incomplete and this hampers food security policy definition, implementation and planning. Crop acreage is one of the fundamental pieces of information needed to quantify food production at the regional or country level. Satellite images can contribute to provide such fundamental information for the implementation of a robust and sustainable agricultural management system and monitor the progress towards the SDGs (Noort, 2017). A satellite-based approach can drastically reduce costs compared to traditional field surveys and can improve efficiency, which opens the possibility to systematically map agricultural resources over large geographical areas in the African continent, as in other parts of the world. Very High spatial Resolution (VHR) images can be used for mapping large geographical areas. Nevertheless, accurately mapping agricultural resources in Africa is a challenging task because of the characteristics of smallholder farms: (i) small plot size (< 2 ha); (ii) irregularly shaped fields with often indistinct boundaries; (iii) strong seasonal variations in surface reflectance; (iv) predominantly rain-fed practices that naturally coincide with high incidence of clouds; (v) high spatiotemporal dynamics.

**Fig. 1:**
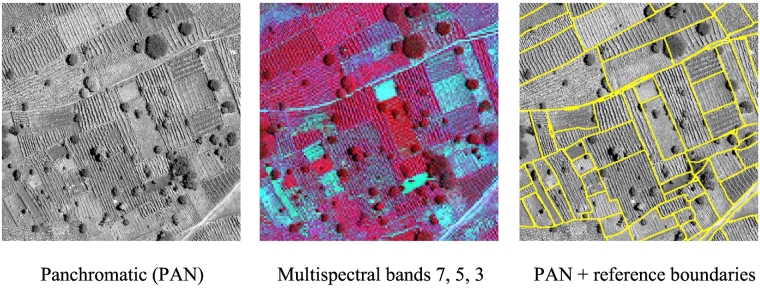
– Subset of a WorldView-3 image acquired over Kofa, Nigeria.

In this paper, we focus on the delineation of agricultural fields from VHR satellite imagery, where a field is an area of land used for agricultural purposes on which a specific crop or a crop mixture is cultivated. This definition coincides with the definition of plot adopted in FAO (2010). We prefer here to use the term field, as more commonly adopted in the literature. Field boundaries are defined as boundaries where a change in crop type, crop mixture or farm management practice takes place, or where two similar cultivations are separated by a natural disruption in the landscape, like a road or a ditch. An accurate delineation of agricultural fields is important because it enables to aggregate crop statistics and yield information at the field level (Rydberg and Borgefors, 2001). Accurate field segments are also useful for further analysis to map crop type, adopting for instance an object-based classification approach (Blaschke, 2010; Zhao et al., 2017). Previous research on field boundary delineation from remote sensing data has mainly focussed on areas characterized by large plots using medium resolution images (Graesser and Ramankutty, 2017; Rydberg and Borgefors, 2001; Yan and Roy, 2014). Automatic delineation of fields in smallholder farms is extremely challenging since boundaries are often not characterized by clearly visible edges, but need to be extracted by detecting changes in the textural and spectral patterns of different cultivations. In these circumstances, standard techniques for edge detection typically fail in achieving the required accuracy. Fig. 1 shows a detail of WorldView-3 image acquired over Kofa, Nigeria, used later in our experimental analysis, illustrating the complex geometrical characteristics of smallholder farm fields.

### 1.1 Background and related work

Edge and contour detection have a long history in image processing and computer vision. The early research focussed on the design of filters for the detection of intensity or colour gradients. The Roberts (Roberts, 1965), Sobel (Duda and Hart, 1973) and Prewitt (Prewitt, 1970) operators use convolutional filters to detect local directional derivatives. Marr and Hildreth (1980) utilise the zero crossings of the Laplacian Gaussian operator. The popular Canny detector also finds the pixels with the highest gradients in their local neighbourhood (Canny, 1986), adding non-maximum suppression and hysteresis thresholding steps. The main problem with these operators is that they only consider the colour and intensity differences between adjacent pixels but cannot tell the textural differences in a larger neighbourhood, which is of fundamental importance for the analysis of agricultural areas from high-resolution imagery.

With the advance of texture analysis techniques, Martin et al. (2004) carefully designed features based on colour, brightness and texture to extract boundary strength with a logistic regression classifier. Ren et al. (2005) introduced a method based on conditional random fields to enforce curvilinear continuity of contours. Their scale-invariant technique is capable of filling short gaps in the detected contours. Arbeláez et al. (2011) developed a technique known as gPb (globalized probability of boundary), by combining multiscale local cues based on colour, brightness and texture with global image information to predict boundary probabilities. The globalisation framework based on spectral clustering allows to connect edge fragments and obtain extended and smooth contours. This is performed by using the local cues computed by oriented gradient operators to define an affinity matrix representing the similarity between pixels. From the affinity matrix, Arbeláez et al. derived a generalised eigenproblem and solve it for a fixed number of eigenvectors, which carry contour information. Local cues and global information are finally combined to obtain the globalized probability of boundary (gPb). In the context of remote sensing, Crommelinck et al. (2017) investigated the use of gPb for the extraction of cadastral boundaries using high resolution Unmanned Aerial Vehicles (UAV) images. Pont-Tuset et al. (2017) proposed a unified approach to hierarchical segmentation and object proposal generation called Multiscale Combinatorial Grouping (MCG). Their main contributions consist of an efficient normalized cut algorithm for the eigenvector computation required for contour globalisation and a grouping algorithm that efficiently explores the combinatorial space of a multiscale hierarchical segmenter. They also propose a faster version named Single-scale Combinatorial Grouping (SCG).

Another research line is investigating the use of trainable models for edge detection, instead of relying on hand-crafted features. Dollar et al. (2006), proposed a boosted edge learning algorithm to train an edge classifier from thousands of simple features. Dollár and Zitnick (2014) propose a supervised learning approach using a structured random forest. Their method is an order of magnitude faster than previously proposed methods, while achieving high accuracy on popular computer vision benchmarks. In contrast to generic edge detection techniques, which aim at detecting any edge in the image, supervised models can be trained to detect specific edges of interests, while discarding irrelevant ones. This task is referred to as *semantic edge detection* or *boundary detection* when the edges of interestes are those separating different object categories (Hariharan et al., 2011).

The most recent wave of (semantic) contour detection algorithms make use of deep learning networks, which have shown remarkable capability in learning high-level data representation for object recognition, image classification and semantic segmentation (pixel-wise classification) (Bertasius et al., 2015; Maninis et al., 2018; Shen et al., 2015; Xie and Tu, 2015; Yang et al., 2016). Among the other deep network typologies, Convolutional Neural Networks (CNNs) became very popular in image analysis because of their capability to learn a hierarchy of spatial features at different layers of the network associated to increasing levels of abstraction, i.e., from raw pixel values to parts of objects (edges and corners), local shapes, up to complex textural patterns (Bergado et al., 2016; Farabet et al., 2013; Krizhevsky et al., 2012; Szegedy et al., 2015). In the remote sensing literature, CNNs have been applied, among others, to scene classification (Cheng et al., 2018b), land-cover or land-use classification (Bergado et al., 2018, Bergado et al., 2016; Fu et al., 2017; Maggiori et al., 2017; Paisitkriangkrai et al., 2016; Volpi and Tuia, 2017), feature extraction and classification of hyperspectral images (Cheng et al., 2018a; Ghamisi et al., 2016; Zhao and Du, 2016), object localization and detection (Chen et al., 2016; Cheng et al., 2016; Long et al., 2017), digital terrain model extraction (Gevaert et al., 2018; Rizaldy et al., 2018), and informal settlement detection (Mboga et al., 2017; Persello and Stein, 2017).

Various architectures of convolutional networks and training strategies have been investigated for object-contour detection on large computer vision data sets. Bertasius et al. (2015) make use of high-level object-related features generated by pre-trained CNNs to predict contours. Their multi-scale deep network architecture consists of five convolutional layers and a bifurcated fully-connected sub-network. Their study shows that without any feature engineering the multi-scale deep learning approach achieves state-of-the-art results in contour detection. Xie and Tu (2015) propose an edge detection algorithm that uses a Fully Convolutional Network (FCN) with multiple side outputs, named holistically-nested architecture, for a deeply supervised training. Their method significantly increases the detection accuracy and reduces, at the same time, the computational cost. Shen et al. (2015) adopt a new loss function, named positive-sharing loss, in which each subclass shares the loss for the whole positive class (contours). Compared to the commonly adopted softmax, their loss function introduces an extra regularized which facilitates to explore more discriminative features. Yang et al., 2016 use an encoder-decoder FCN to detect foreground object contours while suppressing background edges and adopt MCG to generate object proposals.

In the context of urban remote sensing applications, Marmanis et al., (2018) developed a deep CNN-based ensemble model for semantic segmentation, which is explicitly extracting boundaries between regions of different land-cover classes. Their models showed state-of-the-art results on two benchmark data sets of aerial images acquired over urban areas. Volpi and Tuia (2018) introduced a classification strategy based on a multi-task CNN providing both class likelihoods and probability of boundaries. The extracted information is then combined with a spatial regularization framework encoded by a conditional random field model that optimizes the label space across the segmentation hierarchy.

### 1.2 Main contributions

To the best of our knowledge, the use of deep learning techniques for agricultural field delineation from satellite images has not been explored yet. This paper introduces a strategy based on an encoder-decoder FCN and a grouping algorithm to segment fields in smallholder farms from satellite VHR images. The FCN is trained to detect field boundaries discarding irrelevant edges present in the image. The detected sparse contours are then used to extract a hierarchy of closed segments employing the Oriented Watershed Transform (OWT) and iteratively merging adjacent regions based on the average strength of their common boundary (Arbeláez et al., 2011). The final segmentation is obtained by applying the SCG algorithm that efficiently explores the combinatorial space of the segmentation hierarchy to generate accurate field segments (Pont-Tuset et al., 2017; Yang et al., 2016). Our main contributions are:

- The introduction of an automated technique based on a deep FCN and combinatorial grouping to delineate agricultural fields in smallholder farms from VHR images;
- An extensive experimental analysis for two study areas in Nigeria and Mali, which compares the proposed strategy against several computer vision baseline methods;
- The introduction of the boundary-based precision-recall accuracy assessment framework in remote sensing, which tolerates small localisation errors in the detected boundaries.

## 2 Study areas and available data

This section introduces the two study areas and the available data considered in our experimental analysis.

### 2.1 Kofa study area and data

Our first study site is a 3 × 2 km area of intensive but small-scale, rain-fed agricultural production in the Sudano-Sahelian savanna region of northern Nigeria, around the city of Kofa, Bebeji Local Government Area, Kano state. This area can be characterized as having small fields (average 0.22 ha), with only 5% pure crops, and >50% having three or more crops at any moment in the crop season. The farm field landscape is further characterized by many scattered trees. Important crops in this area are sorghum, rice, millet, maize and groundnut. The site was under study by the International Crop Research Institute for the Semi-Arid Tropics (ICRISAT) Nigeria, ICRISAT Mali and the ITC Faculty in the context of the STARS0F^1^ project during 2014–1016.

**Fig. 2:**
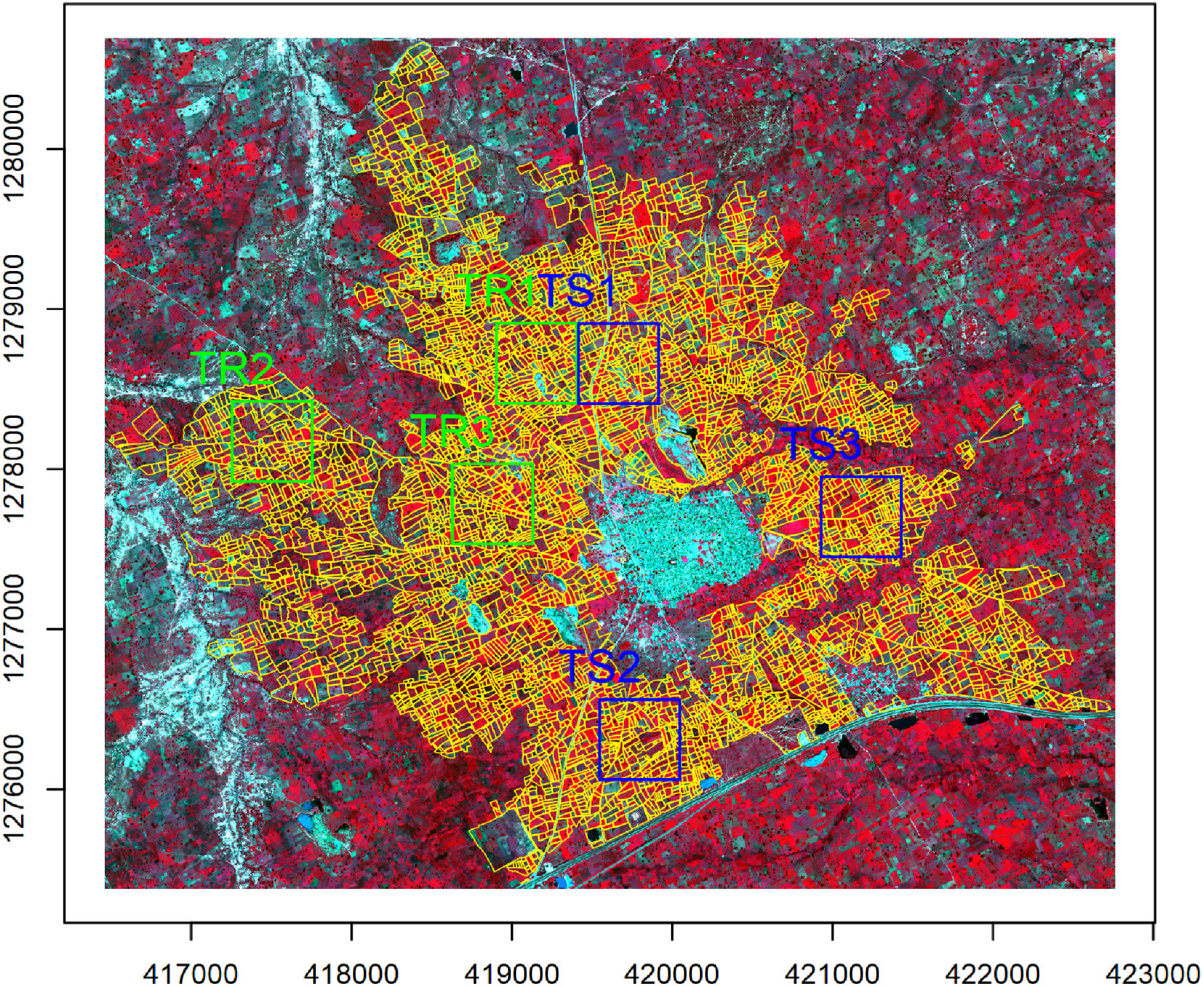
False colour composite (bands 7, 5, 3) of the WorldView-3 image acquired over the Kofa study area on 25 September 2015. (C) 2015 DigitalGlobe, Inc., Longmont CO USA 80503. The image is in WGS 84 UTM zone 32 projection, units are meters. Field polygons and lines are shown in yellow. Training and testing tiles are indicated by the green and blue squares, respectively. (For interpretation of the references to colour in this figure legend, the reader is referred to the web version of this article.)

Field boundary data, comprising over 5000 polygons, were obtained for the year 2015 by ICRISAT Nigeria through an intensive field campaign, funded by the STARS project, using GPS-enabled smartphones and tablets. Using a WorldView-3 image, acquired through satellite tasking over the study site on September 25th 2015, we subsequently corrected that original dataset by human photo-interpretation and expanded it to over 5700 field boundaries, using visual clues from the pan-sharpened image product. The WorldView-3 data contains a panchromatic (PAN) channel at 0.5 m resolution and eight multispectral (MS) bands at 2 m. The product is atmospherically corrected, orthorectified, and co-registered using the STARS project image workflow (Stratoulias et al., 2017). Six tiles of 1000 × 1000 pixels were selected for our experimental analysis (see Fig. 2).

**Fig. 3:**
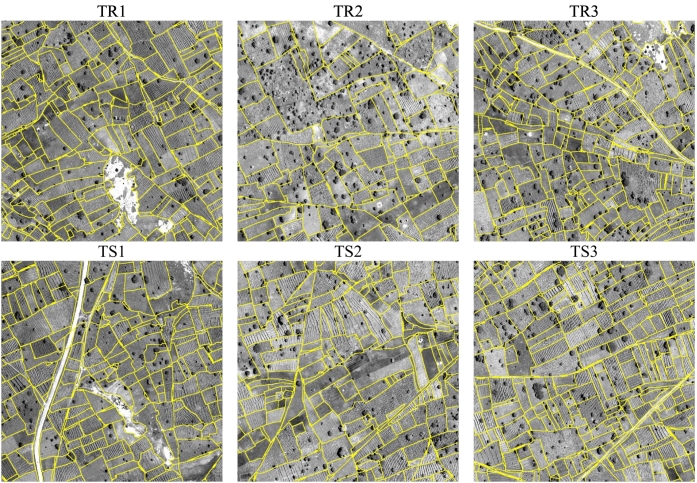
Tiles of the Kofa data set used in the experimental analysis. Field reference boundaries are superimposed over the panchromatic channel.

Tiles TR1–TR3 were used for training and TS1–TS3 for accuracy assessment. We prepared raster images of field boundaries with the grid matching the PAN image. Conversion from vector line boundaries to raster was done in three steps. First, pixels with central point within 1 m from the lines were selected as candidate boundary pixels. Second, morphological thinning was applied. Finally, we applied dilation and closing with a 3 × 3 square. In this way, the raster boundaries had a uniform thickness. Fig. 3 shows the considered six tiles where the field reference boundaries are superimposed over the PAN. In this figure, we display the PAN as it allows one to better appreciate the fine texture represented by the narrow crop rows in the fields than the MS bands.

### 2.2 Sougoumba study area and data

Our second study site is a 6 × 5 km area of small-scale, rain-fed agricultural production in the Sudano-Sahelian savanna region of south-eastern Mali, about 5 km north from the city of Sougoumba, in Sikasso region. This area can be characterized as one with larger fields (average 1.35 ha) than in Kofa, of which a substantial majority have pure crops, and a negligible percentage have three or more crops at any moment in the crop season. The farm field landscape has many scattered trees, of which especially African Baobab (*Adansonia digitata*) and African Shea (*Vitellaria paradoxa*) are valued. Important crops in this area are millet, cotton, sorghum, maize, millet, ground nuts and various garden kitchen vegetables like pumpkin. This site was also under study by ICRISAT Nigeria, ICRISAT Mali and the ITC Faculty in the context of the STARS.

**Fig. 4:**
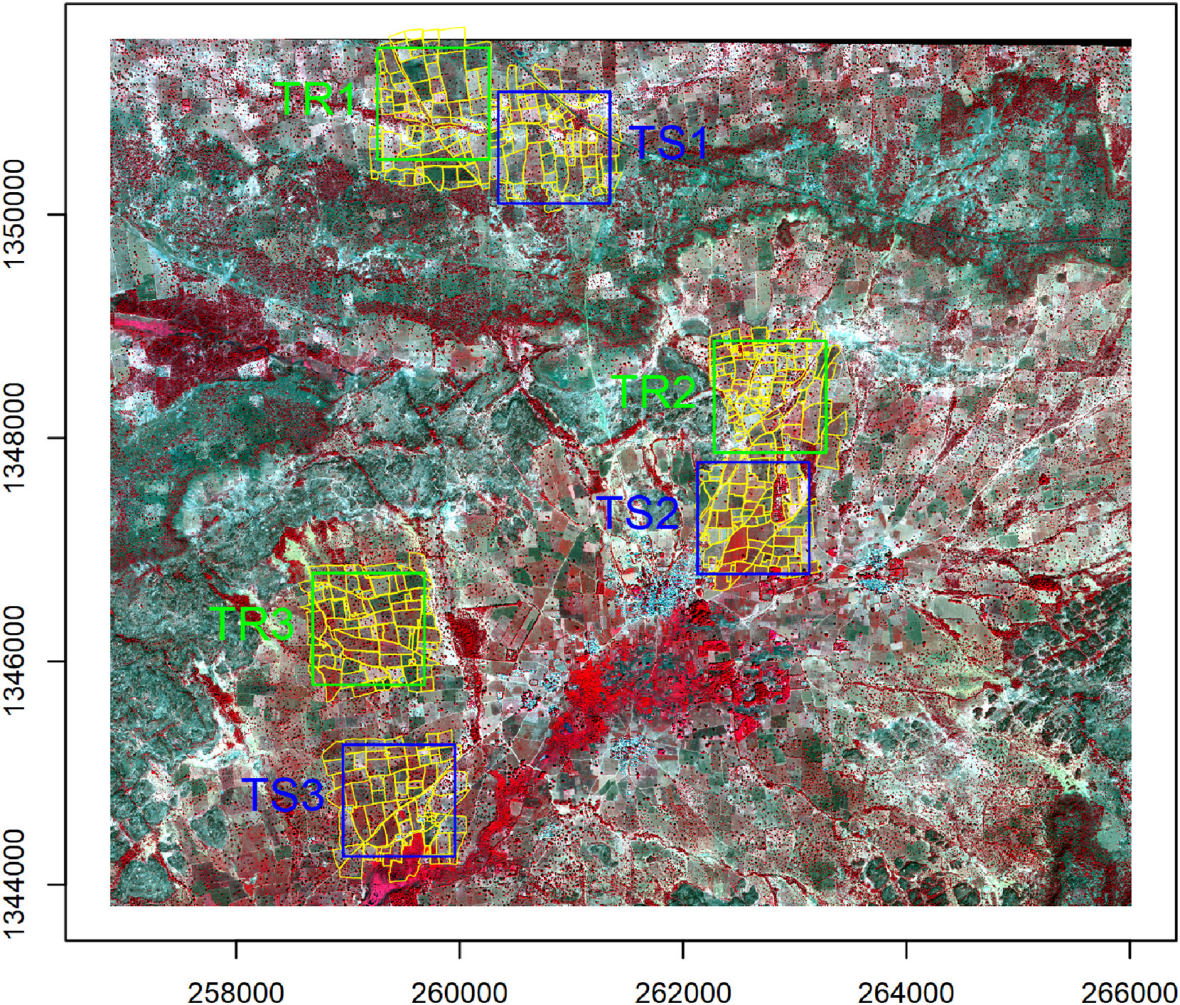
False colour composite (bands 7, 5, 3) of the WorldView-2 image acquired over the Sougoumba study area on 14 November 2014. (C) 2015 DigitalGlobe, Inc., Longmont CO USA 80503. The image is in WGS 84 UTM zone 30 projection, units are meters. Field polygons and lines are shown in yellow. Training and testing tiles are indicated by the green and blue squares, respectively. (For interpretation of the references to colour in this figure legend, the reader is referred to the web version of this article.)

**Fig. 5:**
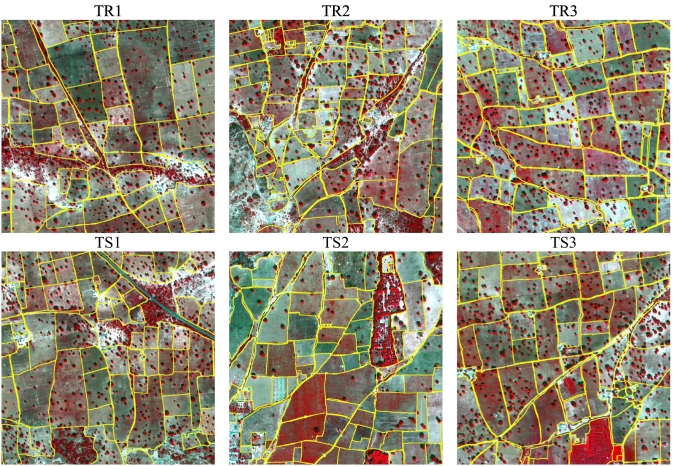
Tiles of the Sougoumba data set used in the experimental analysis. Field reference boundaries are shown in yellow, superimposed over the false colour composite (bands 7, 5, 3). (For interpretation of the references to colour in this figure legend, the reader is referred to the web version of this article.)

Field boundary data, comprising over 500 field polygons, were obtained for the year 2014 by ICRISAT Mali through an intensive field campaign using mobile devices. Using a WorldView-2 image, acquired through satellite tasking over the study site on November 14th 2014, we subsequently verified and corrected that original dataset by human photo-interpretation for the area of interest of this study. The WorldView-2 data contains a panchromatic (PAN) channel at 0.5 m resolution and eight multispectral (MS) bands at 2 m. Six tiles of 2000 × 2000 pixels were selected for the experimental analysis of this study area, with the four times larger size motivated by the larger average field size in Mali (see Fig. 4). Conversion from vector line boundaries to raster was done following the same steps as for the Kofa case. Fig. 5 shows the considered six tiles where the field reference boundaries are superimposed over the false colour composite of MS bands 7, 5, 3. This visualization, using the MS bands instead of the PAN, allows one to better appreciate the spectral differences between fields, which are more prominent in Sougoumba than in Kofa. In contrast, crop rows are much less visible in Sougoumba than in the previous study area.

## 3 Methods

### 3.1 Field boundary detection with a fully convolutional network

The proposed boundary detection strategy takes advantage of the recent success of Fully Convolutional Networks (FCN) for pixel-wise classification (Shelhamer et al., 2017). In contrast to traditional CNNs, which predict one class label per input image, FCNs are designed to infer pixel-wise predictions directly, independently from the size of the input image. In these architectures, the fully connected layers of standard CNNs are usually substituted by deconvolution or unpooling layers that upsample the feature maps learned by the convolutional layers to the resolution of the input image (Badrinarayanan et al., 2017; Noh et al., 2015). An alternative approach is to use no-down-sampling networks employing dilated convolutional filters as in (Persello and Stein, 2017; Yu and Koltun, 2016).

**Fig. 6:**
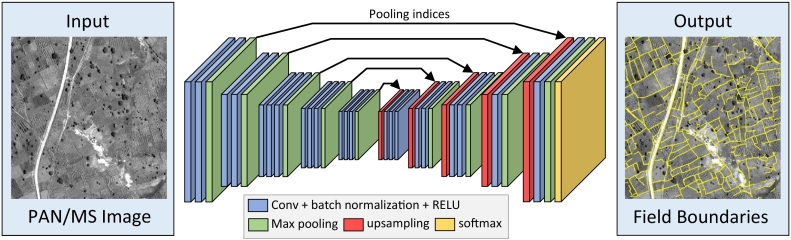
Illustration of the SegNet architecture, adapted from (Badrinarayanan et al., 2017). The network does not employ fully connected layers and is hence only convolutional. The encoder is identical to the VGG-16 architecture. The decoder upsamples its input features using the pooling indices transferred from the corresponding layers in the encoder to produce sparse feature maps. It then performs convolution to densify the feature maps. The final feature maps are fed to a soft-max classifier to predict pixel-wise class probabilities.

We formulate the field boundary detection as a supervised pixel-wise image classification problem to distinguish “boundary” from “non-boundary” pixels, respectively. The classification algorithm is trained to specifically detect field boundaries, therefore performing semantic edge detection. To this aim, we adopt the SegNet architecture (Badrinarayanan et al., 2017), which consists of a deep encoder-decoder fully convolutional network for pixel-wise labelling (Fig. 6). The encoder part of the network is topologically identical to the convolutional layers of the VGG-16 network (Simonyan and Zisserman, 2015), including 13 convolutional layers followed by batch normalization and Rectified Linear Units (RELU), and 5 max-pooling layers, each of them down-sampling the spatial resolution of the input feature maps by a factor two. The architecture is similar to the one used in (Yang et al., 2016), but the fully connected layers are removed. This makes the SegNet encoder significantly smaller and easier to train. The decoder is used to map the low-resolution feature maps learned by the encoder to the full resolution of the input image. Instead of using deconvolution or transposed convolutions, the decoder of SegNet uses pooling indices computed in the corresponding max-pooling layers of the encoder to perform non-linear upsampling. The obtained upsampled maps are sparse and are then convolved with trainable convolutional filters to produce dense feature maps. This procedure eliminates the need for learning to upsample, reducing the number of trainable parameters and improving the accuracy of boundary delineation. The abovementioned characteristics make SegNet well suited for the considered contour detection problem.

SegNet is designed for semantic segmentation of RGB images and therefore accepts only three input channels. We extend the network to take as input the (pan-sharpened) multispectral bands, including additional filters in the input layer to match the number of input channels. The encoder part is initialised with the pre-trained VGG-16 net, except for the additional filters which are randomly initialized. The decoder is also initialized randomly. For training the network, we randomly extract patches from the training tiles to reduce memory consumption. The influence of the patch size on the detection accuracy is analysed in Section 4. We train the network for 300 epochs using the Adam (adaptive moment estimation) optimizer (Kingma and Ba, 2014), which we found more efficient and stable with respect to the choice of the hyper-parameters than the common stochastic gradient descent. Since the “boundary” and “non-boundary” pixels are largely unbalanced, we set the penalty for misclassifying the “boundary” class to be 10 times higher than for the “non-boundary”.

### 3.2 Connecting boundaries and field segment generation

**Fig. 7:**
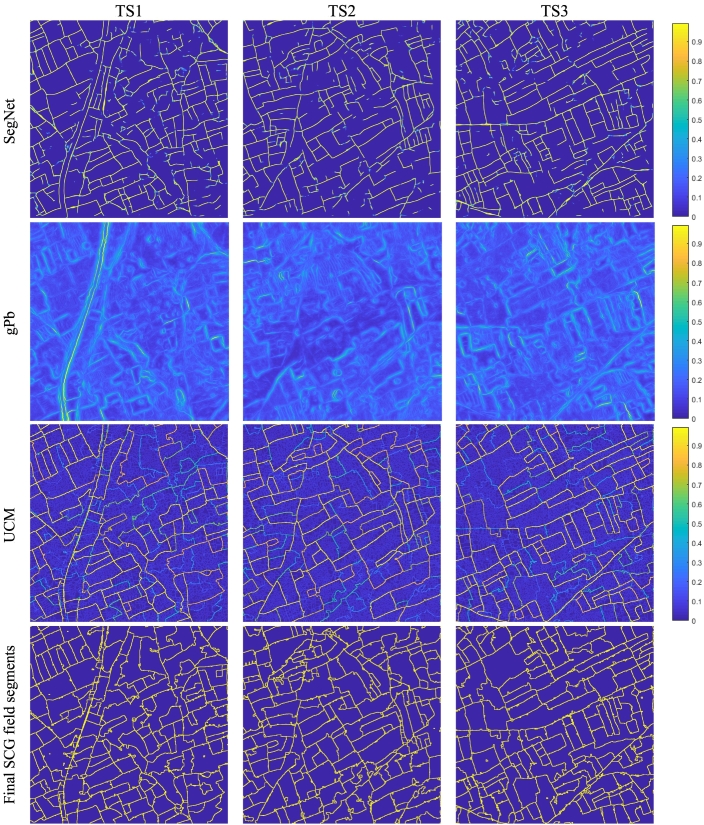
Intermediate and final results of the proposed segmentation strategy on the Kofa test tiles. First row: boundary strength predicted by SegNet. Second row: gPb edge strength. Third row: UCM obtained by applying OWT-UCM to the combined local cues (gPb and SegNet). Forth row: final SCG field segments.

The contour detector based on the binary SegNet classification results in fragmented contours, which do not partition the image into closed segments. As an illustrative example, Fig. 7, first row, shows the boundary strength extracted by SegNet from the Kofa test tiles. One can always recover closed contours from a segmentation in the form of their boundary, but the reverse operation is not trivial. We adopt a two-step technique to recover a hierarchical segmentation from fragmented contours proposed by Arbeláez et al. (2011). The first step consists in applying the OWT to construct the finest set of regions, i.e., an over-segmentation from an oriented contour signal. The second step makes use of an agglomerative clustering procedure to progressively merge the most similar adjacent regions by removing the weakest common boundary based on the average boundary strength. This process results in a hierarchy of regions that can be represented as an Ultrametric Contour Map (UCM), a real-valued image obtained by weighting each boundary by its scale of disappearance. In this work, we linearly combine the gPb detector with our SegNet-based semantic edge detector and obtain a hierarchical segmentation using the OWT-UCM procedure. Fig. 7 shows the gPb contour (second row) and the UCMs (third row) derived from the three Kofa test tiles. We also experiment with a strategy that is applying OWT-UCM directly to SegNet-based contours (excluding gPb).

Based on the obtained UCM, one can derive a segmentation by extracting values greater than a threshold parameter. Varying the threshold using decreasing values from one to zero results in more and more detailed segmentations; zero is the case preserving all edges and therefore corresponds to the over-segmentation with the highest level of detail. However, this approach requires to tune the threshold parameter. In addition, since the segmentation hierarchy is built on low-level local features and does not consider semantic information of objects (crop type and land cover classes in our case), it is possible that the regions obtained from a single level in the UCM hierarchy do not optimally represent complete objects, i.e., agricultural fields (Pont-Tuset et al., 2017). In other terms, complete objects may require to merge regions from different levels in the segmentation hierarchy. For this reason, the final segmentation is obtained by applying the SCG algorithm that globalises local cues using an efficient implementation of normalized cuts and efficiently explores the combinatorial space of the segmentation hierarchy to generate regions that are likely to represent complete fields (Pont-Tuset et al., 2017; Yang et al., 2016). Fig. 7, fourth row, shows the field segments generated by applying SCG to the Kofa test tiles.

### 3.3 Accuracy assessment

We assess the accuracy using the precision-recall framework introduced in (Martin, 2003; Martin et al., 2004), which is generally applied to evaluate the accuracy in computer vision contour detection benchmarks, but is not common in remote sensing. The main idea is to perform a Bipartite Graph Matching (BGM) between edge fragments, named *edgels* (edge elements), extracted from the detected contours and the reference boundaries and then compute Precision (P), Recall (R), and F-measure (F). The precision measures which proportion of detected boundaries are correct. The recall is the proportion of reference boundary elements correctly detected. A measure that combines precision and recall is the F-measure (or F1-score): it is calculated as the harmonic mean of the two. The simple approach to match collocated *edgels* is typically not appropriate, as we wish to be tolerant of small localisation errors along boundaries. The BGM is therefore constructed by a minimum-cost matching algorithm that minimizes weights that depend on both the Euclidean distance and the orientation difference between *edgels*. An *edgel* in the contour (or segmentation) is connected in the graph to *edgels* in the reference only if the Euclidean distance (in pixels) is less than a pre-defined threshold *d*_*max*_. For more information about the matching algorithm, we refer the reader to Martin (2003) and Martin et al. (2004). In our experiments, we adapted the code published by (Pont-Tuset and Marques, 2016), which allowed us to calculate summary measurements and precision-recall (PR) curves. In computer vision benchmarks, the value of *d*_max_ is set proportional to the length of the image diagonal, e.g., 0.0075 of the image diagonal is the default choice in the provided code (0.01 for the PASCAL segmentation challenge). In remote sensing, *d*_*max*_ can be set according to a tolerable ground localization error knowing the spatial resolution of the input image. In our experimental analysis, we adopt two values for the tolerance buffer: 5 and 10 pixels. Considering the 0.5 m spatial resolution of our VHR images, the buffer values account for 2.5 m and 5 m ground positional tolerance, respectively.

## 4 Experimental analysis

### 4.1 Experimental set up

This section describes how experiments are conducted and introduces the abbreviations used hereafter for the considered techniques. In our experimental analysis, we compare the performance of several state-of-the-art contour detection and hierarchical segmentation techniques on the test tiles of the two considered data sets. The shallow techniques (i.e., not based on deep learning) gPb-owt-ucm, SCG and MCG are applied to the pan-sharpened multispectral bands 7, 5, 2 of the WorldView-2/3 images. Pansharpening is performed using the Gram-Schmidt algorithm. SCG and MCG use the pre-trained structured-forest contour detector (Dollár and Zitnick, 2014) for the extraction of the low-level cues while gPb-owt-ucm uses hand-crafted multiscale local cues based on colour, brightness and texture. In the case of gPb-owt-ucm, the final segmentation level is obtained by tuning the threshold parameter on the UCM derived from the training tiles. SCG-ucm and MCG-ucm refer to the corresponding techniques extracting the UCM segmentation hierarchies before applying the combinatorial grouping procedure to obtain the final object proposals. We also investigate a contour detector based on the six-layer FCN with dilated kernels (FCN-DK6) introduced in Persello and Stein (2017) using the PAN band as input. The max-pooling layers were removed to avoid smoothing of contour lines. We combined FCN-DK6 with SCG to derive an UCM (FCN-DK6-SCG-ucm) and field segments (FCN-DK6-SCG).

**Fig. 8:**
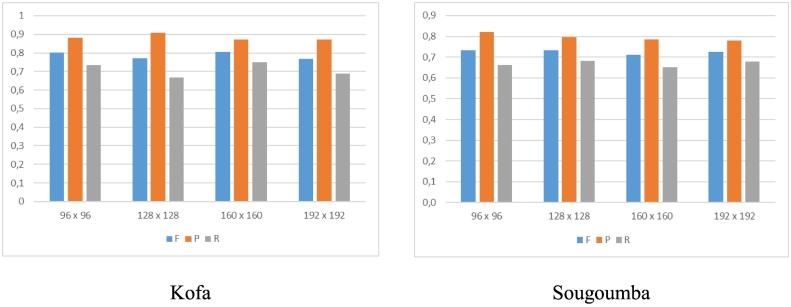
Detection accuracy of SegNet-W-MS varying the patch size. Accuracies are calculated using *d*_*max*_ = *10.*

We applied SegNet to the PAN channel, trained in one case without class re-weighting and in a second case adopting a 10 time higher penalty for the “boundary” class (SegNet-W). We also applied the modified SegNet to the pan-sharpened MS bands with class re-weighting (SegNet-W-MS). In this case, seven MS bands are used as input (bands 2 to 8); the first channel is excluded because it is strongly affected by atmospheric scattering. The network is trained using 6000 image patches randomly extracted from the training tiles. We performed preliminary tests varying the patch size for training SegNet-W-MS, i.e., 96 × 96, 128 × 128, 160 × 160, 192 × 192 pixels. The obtained results, reported in Fig. 8, show little variation on both data sets. We consequently fixed the patch size to 96 × 96 pixels, which is large enough to capture the relevant contextual information but limits the memory footprint and the training time with respect to larger patches. The networks are trained with the Adam optimizer using a learning rate of 0.001, also called step size in (Kingma and Ba, 2014), which is adaptively adjusted by using exponential decay rates β_1_ = 0.9 and β_2_ = 0.999 for the moment estimates, respectively. A batch size of 32 is used, running the optimizer for 300 epochs, i.e., a total of 56,400 iterations. The weights are initialized using a normal distribution as proposed in (He et al., 2015).

We then combined SegNet-W-MS with the OWT-UCM and SCG algorithms to obtain a hierarchical segmentation (SegNet-W-MS-SCG-ucm) and the final field segments (SegNet-W-MS-SCG). We also linearly combined the local cues extracted by gPb with SegNet-W-MS to derive the hierarchical segmentation (SegNet-W-MS-gPb-SCG-ucm) and field segments (SegNet-W-MS-gPb-SCG). SegNet-W-MS-SCG and SegNet-W-MS-gPb-SCG represent two variants of the proposed approach.

### 4.2 Results on the Kofa test site

**Fig. 9:**
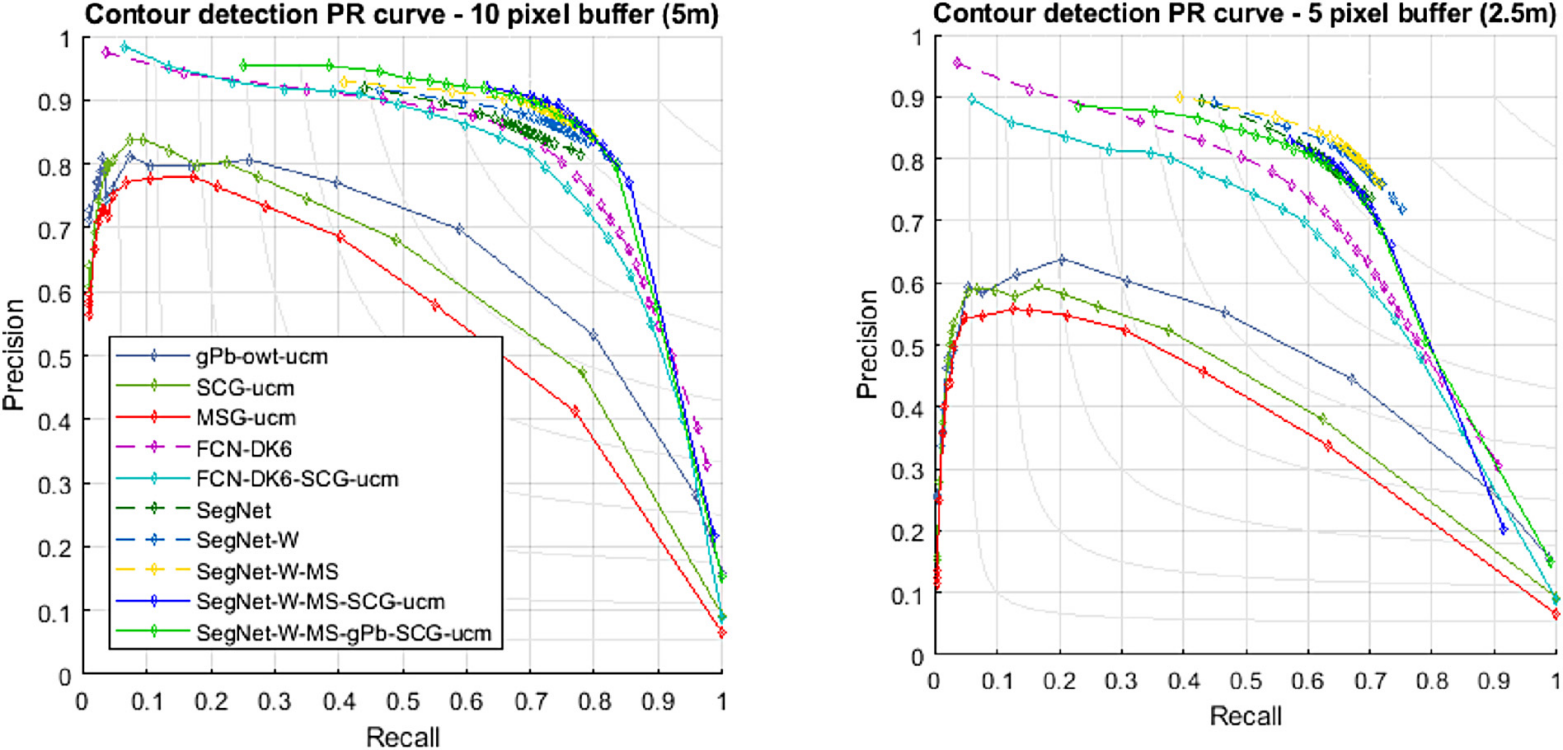
Precision-recall curves obtained for the Kofa test tiles. The solid curves represent result of segmentation algorithms, while dashed lines refer to contour detectors.

**Fig. 10:**
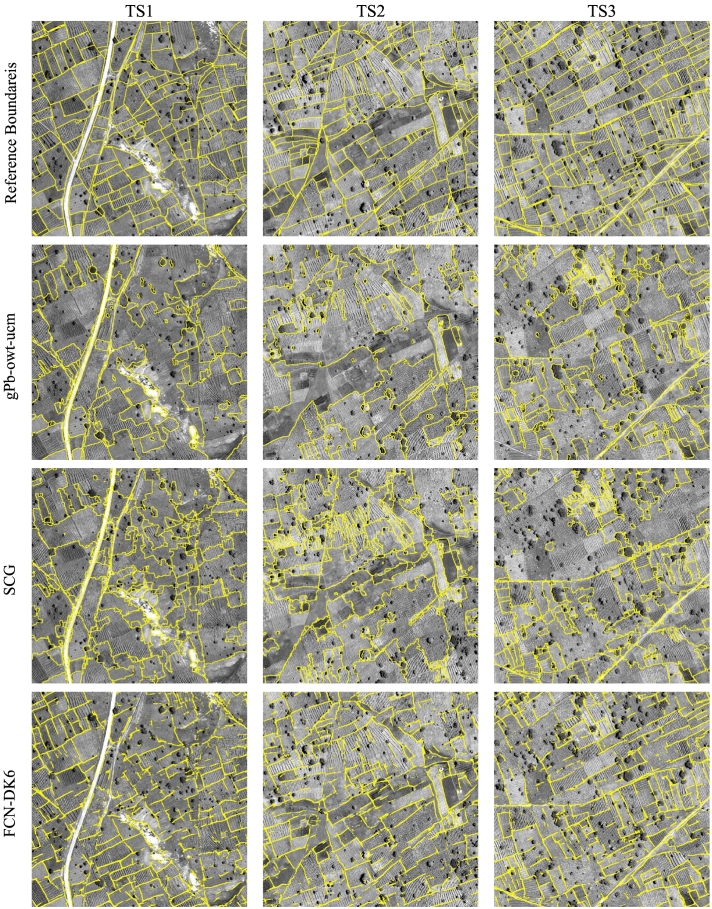

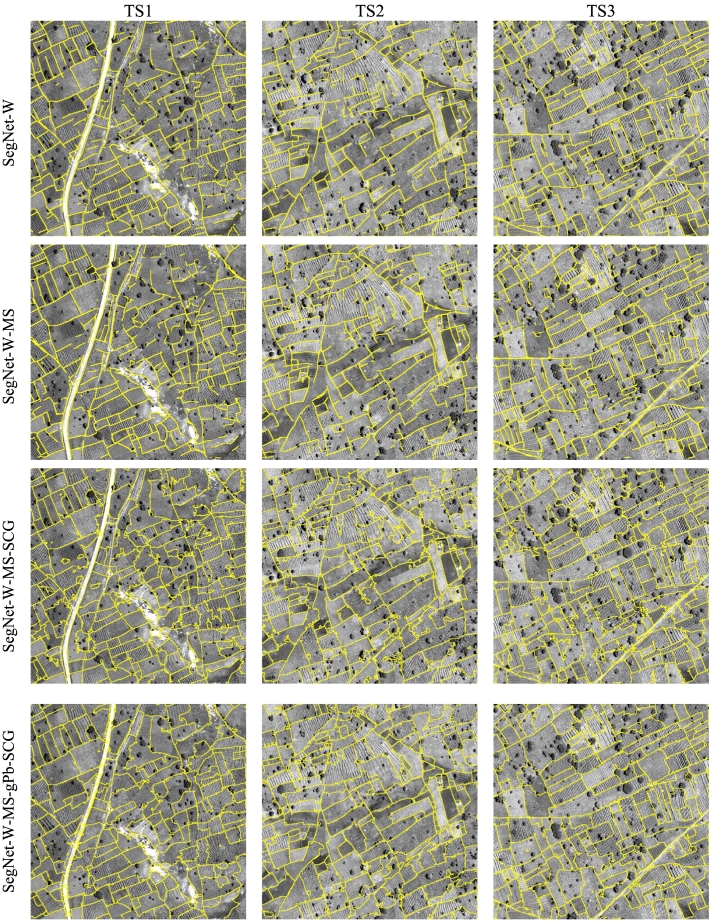
Detection maps of selected contour and segmentation algorithms on the Kofa test tiles.

Table 1, Table 2 report the accuracies obtained on the three Kofa test tiles using *d*_*max*_ of 10 and 5 pixels, respectively. Fig. 9 reports the PR curves considering the two buffer sizes. These curves show different PR trade-offs by varying the threshold applied to the boundary strength or the UCM hierarchies. The standard shallow techniques, gPb-owt-ucm, SCG, and MCG perform poorly in this complex segmentation task. Among the three algorithms, gPb-owt-ucm results in the highest precision, showing a good transferability of the carefully engineered intensity and textural features from natural images to satellite data. From the PR curves, we notice that the hierarchical segmentation extracted by gPb-owt-ucm offers better solutions than SCG and MCG for most of the threshold values. In contrast to the results obtained on computer vision benchmarks, MCG does not provide more accurate field segments than SCG. Fig. 10 shows the obtained field detection/segmentation maps for most of the considered techniques. The results of gPb-owt-ucm and SCG are visually not satisfactory, missing several field boundaries and delineating smaller irrelevant elements like trees and crop row structures within fields.

**Table 1:** – Contour accuracy assessment on the Kofa test tiles (*d*_*max*_ = 10 px, i.e., 5 m).

Method	Tile TS1			Tile TS2			Tile TS3		
	F	P	R	F	P	R	F	P	R
gPb-owt-ucm	0.663	0.717	0.617	0.577	0.663	0.510	0.668	0.709	0.631
SCG	0.651	0.594	0.719	0.602	0.553	0.661	0.631	0.647	0.617
MCG	0.528	0.380	0.866	0.542	0.420	0.763	0.573	0.475	0.722
FCN-DK6	0.736	0.700	0.776	0.772	0.757	0.788	0.816	0.819	0.812
FCN-DK6-SCG	0.585	0.705	0.501	0.569	0.739	0.463	0.618	0.811	0.499
SegNet	0.758	0.822	0.702	0.748	0.828	0.683	0.787	0.906	0.695
SegNet-W	0.790	0.826	0.757	0.780	0.847	0.723	0.813	0.909	0.736
SegNet-W-MS	**0.801**	**0.846**	0.760	**0.782**	**0.871**	0.709	0.818	**0.925**	0.733
SegNet-W-MS-SCG	0.769	0.677	**0.890**	0.778	0.710	**0.861**	0.829	0.782	**0.882**
SegNet-W-MS-gPb-SCG	0.786	0.730	0.852	0.781	0.721	0.851	**0.830**	0.823	0.836

Bold numbers indicate the highest metric across the considered techniques.

**Table 2:** – Contour accuracy assessment on the Kofa test tiles (*d*_*max*_ = 5 px, i.e., 2.5 m).

Method	Tile TS1			Tile TS2			Tile TS3		
	F	P	R	F	P	R	F	P	R
gPb-owt-ucm	0.547	0.456	0.684	0.501	0.418	0.625	0.554	0.459	0.699
SCG	0.502	0.458	0.554	0.470	0.431	0.516	0.482	0.494	0.471
MCG	0.443	0.318	0.726	0.440	0.341	0.620	0.457	0.379	0.576
FCN-DK6	0.620	0.590	0.654	0.666	0.652	0.679	0.708	0.711	0.705
FCN-DK6-SCG	0.488	0.587	0.417	0.463	0.601	0.377	0.514	0.675	0.415
SegNet	0.680	0.738	0.630	0.691	0.765	0.630	0.741	0.854	0.655
SegNet-W	0.711	0.744	0.682	**0.726**	0.789	0.673	**0.763**	0.853	0.691
SegNet-W-MS	**0.714**	**0.754**	0.677	0.724	**0.807**	0.656	0.762	**0.862**	0.683
SegNet-W-MS-SCG	0.664	0.584	**0.768**	0.671	0.612	**0.742**	0.702	0.663	**0.747**
SegNet-W-MS-gPb-SCG	0.681	0.632	0.738	0.681	0.628	**0.742**	0.711	0.706	0.717

Bold numbers indicate the highest metric across the considered techniques.

Deep learning techniques perform considerably better on all test tiles. Taking advantage of the available training data, supervised FCNs are capable of learning to detect field boundaries, discarding irrelevant edges present in the image. SegNet provides generally more precise contours than the shallower FCN-DK6 network, but with a lower recall rate. When the accuracy is assessed with 5 m tolerance, the F-score of SegNet is higher than FCN-DK6 on tile TS1, but lower on TS2 and TS3. When the small buffer of 2.5 m is applied, the F-score of SegNet becomes consistently higher than FCN-DK6 on all three tiles. The use of higher penalty for the “boundary” class further improves the result of SegNet. In Fig. 10 we can observe that SegNet-W extracts more sharp and precise field edges, whereas the contours extracted by FCN-DK6 are more noisy. Both techniques result in fragmented contour lines with several gaps. The use of spectral information from SegNet-W-MS offers a small advantage over SegNet-W. The precision attained by SegNet-W-MS is generally slightly higher than SegNet-W, but the F-scores show little or no improvement. The obtained maps appear visually very similar.The combination of SegNet-W-MS with SCG allows one to properly connect the fragmented contours extracted by the deep network, resulting in accurate closed regions representing agricultural fields. Both the proposed SegNet-W-MS-SCG and SegNet-W-MS-gPb-SCG result in higher recall rates, at the expenses of lower precision. The PR curves computed with 5 m tolerance show that SegNet-W-MS-gPb-SCG and SegNet-W-MS-SCG perform slightly better than SegNet-W. With the smaller spatial tolerance value, which favours more precise outputs, SegNet-W-MS attains higher accuracy. The combination of the gPb hand-crafted local cues with SegNet-W-MS, i.e., SegNet-W-MS-gPb-SCG results in higher precision than SegNet-W-MS-SCG. Moreover, from the maps, it visually appears that SegNet-W-MS-gPb-SCG provides in many cases more regular connections between the fragments extracted by the deep network (see e.g., the central part of TS2).

### 4.3 Results on the Sougoumba test site

**Fig. 11:**
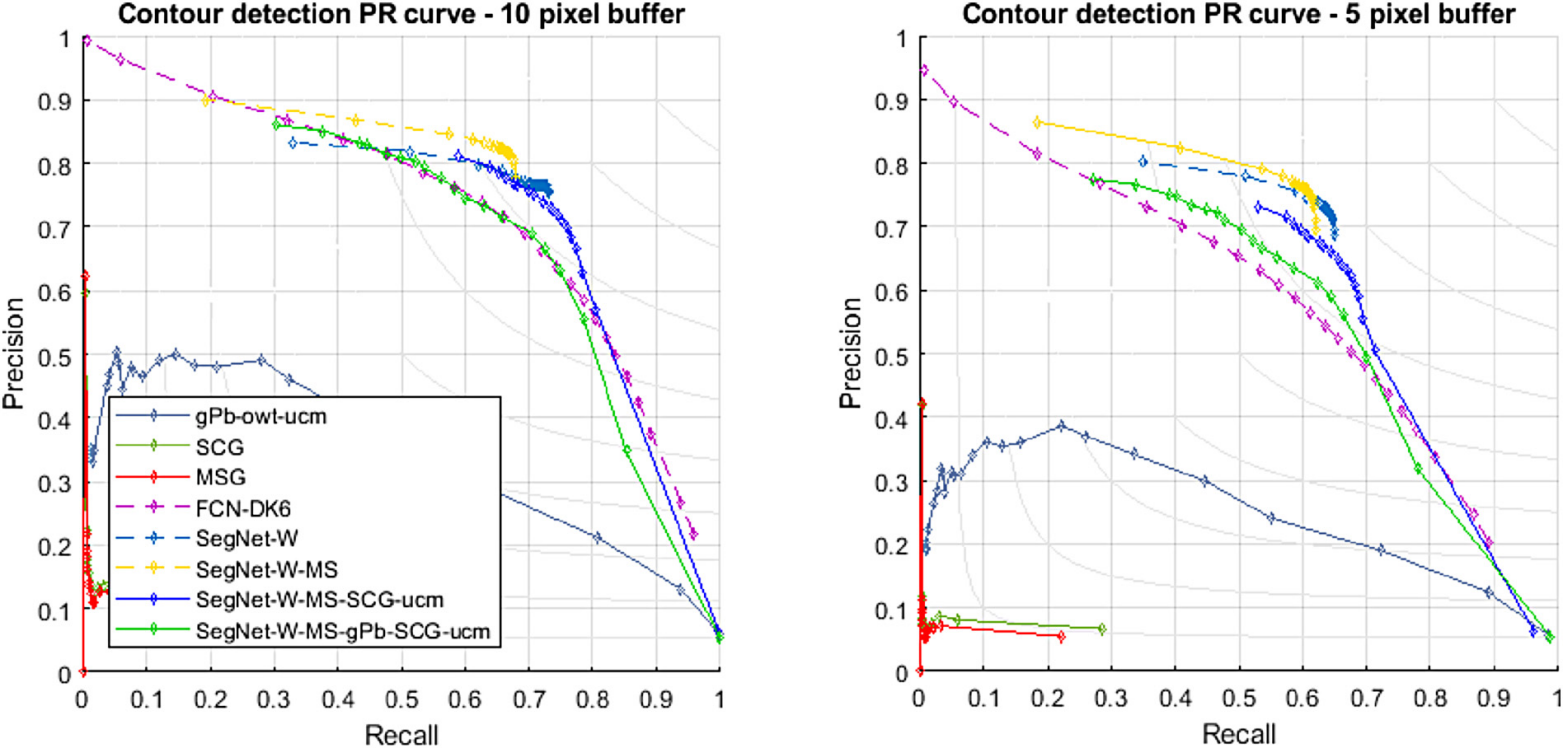
Precision-recall curves obtained for the Sougoumba test tiles. The solid curves represent result of segmentation algorithms, while dashed lines refer to contour detectors.

**Fig. 12:**
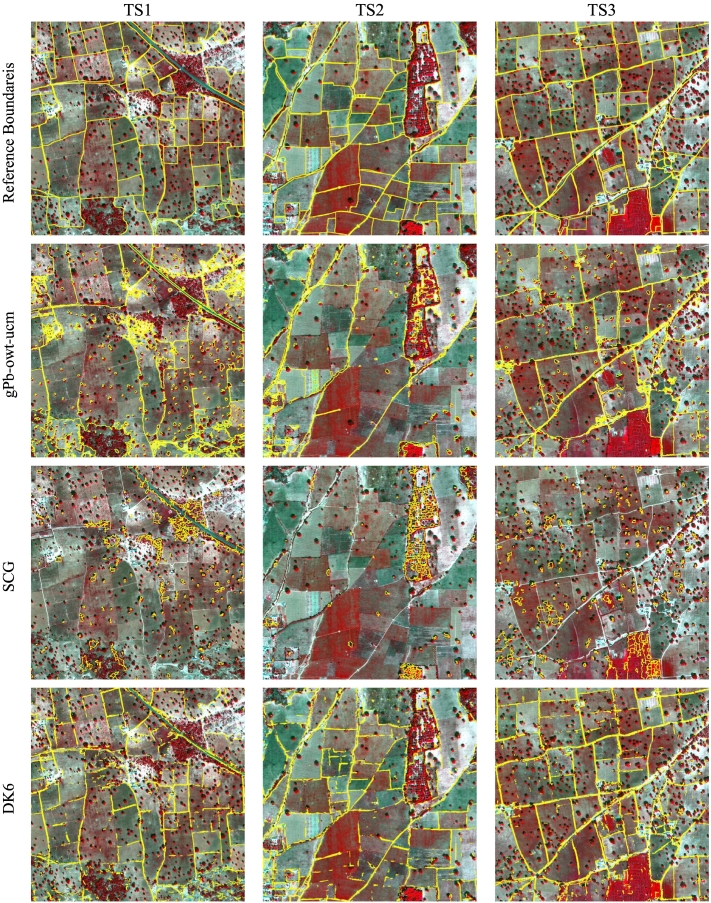

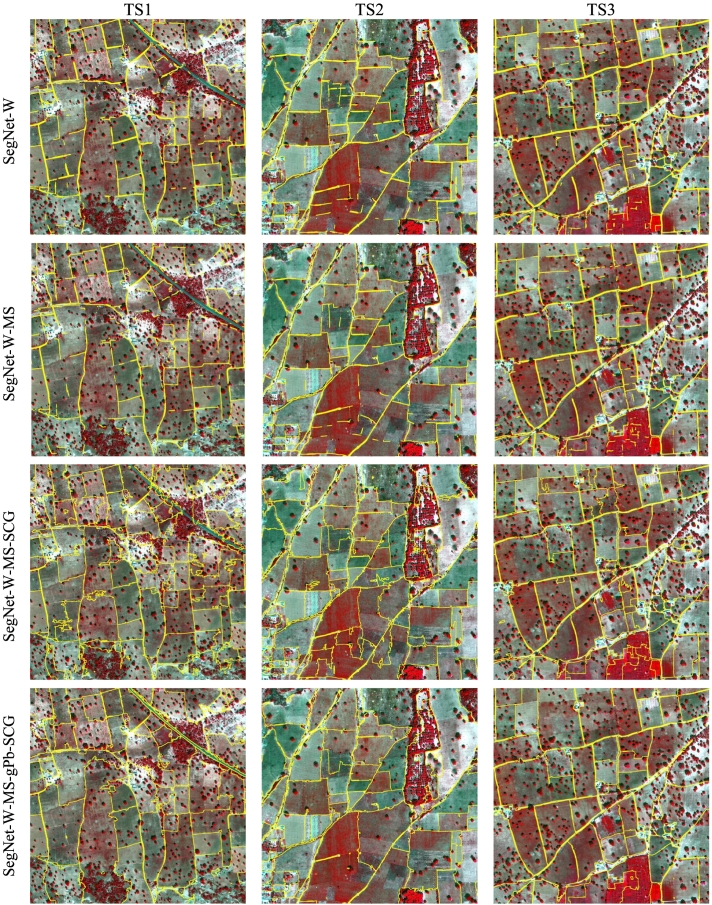
Detection maps of selected contour and segmentation algorithms on the Sougoumba test tiles.

We applied most of the considered techniques also to the Sougoumba test site. We excluded FCN-DK6-SCG, because of the poor performance observed in the Kofa study area, and SegNet, as the advantage of the class re-weighted version SegNet-W has been demonstrated before. The obtained numerical accuracies are reported in Table 3, Table 4. Fig. 11 shows the corresponding PR curves. As evident from the lower accuracies, the Sougoumba data set is more challenging than Kofa, especially tiles TS1 and TS2. The three shallow algorithms have poor performances. Also in this case, gPb-owt-ucm provide better results compared to SCG and MCG, which local cues extracted by the pre-trained structured-forest detector are not transferrable to this complex data set. In contrast, the gPb local cues can provide meaningful information. The poor segmentations are visible in the maps reported in Fig. 12.

**Table 3:** – Contour accuracy assessment on the Sougoumba data set (*d*_*max*_ = 10 px, i.e., 5 m).

Method	Tile TS1			Tile TS2			Tile TS3		
	F	P	R	F	P	R	F	P	R
gPb-owt-ucm	0.359	0.259	0.586	0.393	0.386	0.401	0.545	0.482	0.627
SCG	0.135	0.159	0.117	0.070	0.107	0.053	0.138	0.177	0.113
MCG	0.134	0.138	0.131	0.079	0.106	0.063	0.145	0.160	0.132
FCN-DK6	0.638	0.605	0.675	0.674	0.681	0.666	0.752	0.699	0.815
SegNet-W	0.704	0.733	0.678	**0.706**	0.776	0.649	**0.790**	0.783	0.798
SegNet-W-MS	**0.720**	**0.814**	0.646	0.692	**0.828**	0.595	0.780	**0.820**	0.744
SegNet-W-MS-SCG	0.603	0.478	**0.814**	0.658	0.581	**0.758**	0.749	0.677	**0.839**
SegNet-W-MS-gPb-SCG	0.652	0.584	0.737	0.669	0.666	0.673	0.749	0.749	0.749

Bold numbers indicate the highest metric across the considered techniques.

**Table 4:** – Contour accuracy assessment on the Sougoumba data set (*d*_*max*_ = 5 px, i.e., 2.5 m).

Method	Tile TS1			Tile TS2			Tile TS3		
	F	P	R	F	P	R	F	P	R
gPb-owt-ucm	0.300	0.216	0.489	0.326	0.320	0.333	0.458	0.405	0.528
SCG	0.078	0.092	0.068	0.035	0.053	0.026	0.087	0.111	0.071
MCG	0.074	0.076	0.072	0.038	0.050	0.030	0.094	0.103	0.086
FCN-DK6	0.550	0.521	0.582	0.567	0.574	0.561	0.640	0.594	0.693
SegNet-W	0.659	0.686	0.635	**0.651**	0.715	0.597	0.718	0.711	0.725
SegNet-W-MS	**0.670**	**0.757**	0.601	0.629	**0.752**	0.541	**0.720**	**0.756**	0.686
SegNet-W-MS-SCG	0.540	0.429	**0.729**	0.564	0.498	**0.651**	0.674	0.609	**0.754**
SegNet-W-MS-gPb-SCG	0.587	0.526	0.664	0.579	0.576	0.582	0.672	0.673	0.672

Bold numbers indicate the highest metric across the considered techniques.

FCN-based techniques significantly outperform shallow techniques also on this second data set. SegNet-W provides more accurate results than FCN-DK6, with generally higher precision and F-score. SegNet-W-MS, exploiting also the spectral information, consistently improves the precision with respect to the boundaries detected by SegNet-W. On this data set, the spectral information provides more useful information as compared to the Kofa site. From the detection maps, we can observe the high precision of the boundaries by SegNet-W-MS, while several boundaries remain undetected, as confirmed by the lower recall rate.

The combination of the FCN-detected boundaries with SCG properly connects the fragmented boundaries, generating fairly accurate field segments. Both SegNet-W-MS-SCG and SegNet-W-MS-gPb-SCG result in high recall rates, but also lower precision compared to SegNet-W-MS. In agreement with previous observations, a visual inspection of the maps suggests that SegNet-W-MS-gPb-SCG provides in many cases more regular contours than those produced by the SegNet-W-MS-SCG counterpart.

## 5 Discussion

This study proposes a deep learning based approach to delineate agricultural fields in smallholder farms using VHR satellite images. The effectiveness of the approach has been investigated on two study areas situated in Nigeria and Mali, showing dissimilar characteristics. The first study area, situated in Kofa, Nigeria, is characterized by small fields with irregular shapes, most of them cultivated with a mixed-cropping system. The narrow crop rows are well captured by the 0.5 m resolution PAN channel, which allows the deep FCN to effectively learn to detect the transition in the textural patterns associated with the field boundaries. The second study area, in Sougoumba, Mali, has larger fields, which are on average six times larger than in Kofa, mainly cultivated with a single crop. Crop rows are less evident than in Kofa, textural patterns are less prominent, but the spectral information is relevant to distinguish between the different crop types. In both study areas, field boundaries are often indistinct or vaguely visible and the landscape is characterized by many scattered trees. Despite the complexity of the task and the landscapes, the proposed deep FCN-based techniques attained remarkable accuracies in both cases, significantly outperforming traditional techniques.

**Fig. 13:**
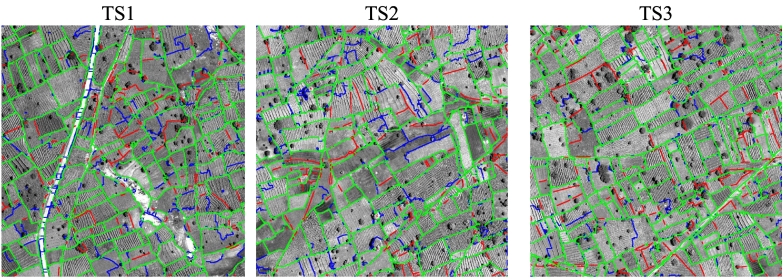
Error maps obtained by the proposed method SegNet-W-gPb-SCG on the Kofa test tiles using a buffer of 10 pixels. Colour map: 1) Green - correct boundary; 2) Red - missed boundary; 3) Blue - false boundary. (For interpretation of the references to colour in this figure legend, the reader is referred to the web version of this article.)

**Fig. 14:**
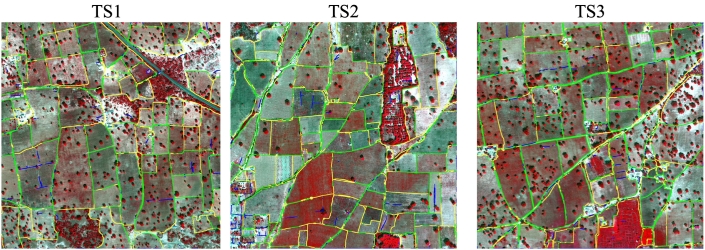
Error maps obtained by the proposed method SegNet-W-gPb-SCG on the Sougoumba test site using a buffer of 10 pixels. Colour map: 1) Green - correct boundary; 2) Yellow - missed boundary; 3) Blue - false boundary. (For interpretation of the references to colour in this figure legend, the reader is referred to the web version of this article.)

Fig. 13 shows the error maps derived by the proposed SegNet-W-gPb-SCG method on the Kofa test site. The large majority of the boundaries are correctly detected, while a few are missed or incorrect, see e.g., several false boundary fragments crossing the large road on the left side of tile TS1. Fig. 14 shows the error maps for the Sougoumba site. Interestingly, the proposed algorithm is not significantly affected by the presence of the many trees and their shadows in the considered areas. In several cases, the field boundary passing close to or under the tree crown is correctly identified. In Fig. 13, tile TS2, some false boundaries are detected in the central part of the tile. However, according to a visual inspection, some of these boundaries look plausible and could have been missed in the reference data collection.

It is worth noting that the reference data could also miss or contain incorrect or imprecise boundaries, as reference data collection is not without challenges. In the field surveys, measurements with (differential) GPS do not scale well and bring accessibility problems due to presence of natural vegetation and terrain topology, hence reducing data consistency. Alternatively, human photo-interpretation scales better, does not have accessibility issues, but in some cases may fail to pick up boundary clues that an in situ visit will bring to the fore. Regardless of the choice, we need to acknowledge that less intense farm practices often cause field boundaries not to be crisply linear, but rather as transition zones where crop plant density approaches zero eventually. This brings a level of arbitrariness in positioning a linear boundary. Generally, the photo-interpreters were instructed to delineate a zone where crop plants from the single field at hand were still expected to grow (or have grown) and not be outnumbered by crop plants from a neighbouring field. Where natural vegetation bordered a field, the edge of its closed canopy was suggested as field boundary, except for cases where seemingly straight field edges were interrupted by larger tree canopies, in which case the interpreter was advised to digitize a (continued) straight field edge.

At a conceptual level, uncertainty may pertain to the ambiguity associated to the actual presence of a boundary (*existential uncertainty*); or the ambiguity of the exact location of the boundary (*positional or extensional uncertainty*) (Lucieer and Stein, 2002). While the correct existence of a boundary is assessed by our validation strategy according to the reference data, the positional uncertainty is explicitly considered in the proposed accuracy assessment framework by tolerating small localization errors. Our experiments are conducted tolerating ground localization errors up to 2.5 and 5 m in the accuracy assessment.

The obtained experimental results reveal the poor performance of the SCG and MCG algorithms based on the pre-trained structure forests predictor. In contrast to the results reported on computer vision benchmarks (Pont-Tuset et al., 2017; Yang et al., 2016), such a model does not generalize well to the considered task. The gPb detector, however, provides good results as a generic edge detector capable to capture small and detailed edges in the image, offering complementary information with respect to the SegNet-based semantic edge detector, specialized in field boundaries. The SegNet architecture, and its modification accepting multispectral images as input, revealed effective in precisely detecting the thin features associated to field boundaries. Thanks to a deeper structure and the use of pooling indices in the decoder part, SegNet proved better suited than the FCN-DK6 architecture. The use of spectral information from the pan-sharpened MS bands offers a small advantage over the network using only the PAN. However, especially for the Kofa data set, this advantage is limited. This is probably due to the fact that in Kofa, the field boundaries can be identified mostly by the transitions of the different textural patterns, which can be properly captured from the panchromatic band, while the spectral information plays a less relevant role. A slightly more significant advantage is observed on the Sougoumba data set, especially in term of precision. The proposed technique based on the combination of SegNet with the SCG combinatorial grouping algorithm, results in an effective strategy to connect fragmented boundaries and obtain connected contours, i.e., field segments. This approach does not require the selection of a threshold or any other user-defined parameter. The two variants SegNet-W-MS-SCG and SegNet-W-MS-gPb-SCG show similar results. However, the version using also the gPb local cues together with the contour extracted by SegNet (SegNet-W-MS-gPb-SCG), generally provides more precise field delineations with smoother contour lines.

## 6 Conclusion

This paper proposes a contour delineation technique based on a deep fully convolutional network and a grouping algorithm to produce a segmentation delineating agricultural fields in smallholder farms. The experimental analysis conducted, using WorldView-2 and 3 images acquired over two study areas, shows promising results. The proposed technique compares favourably against state-of-the-art computer vision contour detection algorithms in terms of the accuracy assessed through the precision-recall framework. A visual inspection of the obtained segmentation results allows us to observe fairly accurate field delineations which are close to human photo-interpretation level. These results show that the proposed automated field delineation method could facilitate the extraction of cadastral boundaries and be incorporated into an object-based image analysis (OBIA) framework for accurate crop type classification. In future studies, we will investigate how to properly integrate the hierarchical segmentation into an end-to-end framework for crop type mapping. Other aspects that need to be further investigated are the use of multi-temporal data and the fusion of panchromatic and multispectral bands within a multiscale contour detection technique.

## Footnotes

1 Available online: http://www.stars-project.org/en/ (accessed October 2018)
